# Biocomputational Assessment of Natural Compounds as a Potent Inhibitor to Quorum Sensors in *Ralstonia solanacearum*

**DOI:** 10.3390/molecules27093034

**Published:** 2022-05-09

**Authors:** Sunil Kumar, Khurshid Ahmad, Santosh Kumar Behera, Dipak T. Nagrale, Anurag Chaurasia, Manoj Kumar Yadav, Sneha Murmu, Yachana Jha, Mahendra Vikram Singh Rajawat, Deepti Malviya, Udai B. Singh, Raja Shankar, Minaketan Tripathy, Harsh Vardhan Singh

**Affiliations:** 1 ICAR-National Bureau of Agriculturally Important Microorganisms, Mau 275103, India; ahmadk@ynu.ac.kr (K.A.); rajawat.mvs@gmail.com (M.V.S.R.); deeptimalviya77@gmail.com (D.M.); nbaimudai@gmail.com (U.B.S.); 2 ICAR-Indian Agricultural Statistics Research Institute, New Delhi 110012, India; murmu.sneha22@gmail.com; 3 National Institute of Pharmaceutical Education and Research, Ahmedabad 382355, India; bioinfo.santosh@gmail.com; 4 ICAR-Central Institute for Cotton Research, Nagpur 440010, India; dip29unique@gmail.com; 5 ICAR-Indian Institute of Vegetable Research, Varanasi 221305, India; govtofindia.icar@gmail.com; 6 Department of Bioinformatics, SRM University, Sonepat 131029, India; manojiids@gmail.com; 7 N. V. Patel College of Pure and Applied Sciences, S.P. University, Anand 388315, India; yachanajha@ymail.com; 8 ICAR-IIHR, Hessaraghatta Lake Post, Bengaluru 560089, India; rajascientists@gmail.com; 9 Department of Pharmacy, Sitaram Kashyap College of Pharmacy, Rahod 495556, India; minaketantripathy@gmail.com

**Keywords:** quorum-sensing, PhcA and PhcR, molecular docking, natural compounds, *Ralstonia solanacearum*

## Abstract

*Ralstonia solanacearum* is among the most damaging bacterial phytopathogens with a wide number of hosts and a broad geographic distribution worldwide. The pathway of phenotype conversion (Phc) is operated by quorum-sensing signals and modulated through the (R)-methyl 3-hydroxypalmitate (3-OH PAME) in *R. solanacearum*. However, the molecular structures of the Phc pathway components are not yet established, and the structural consequences of 3-OH PAME on quorum sensing are not well studied. In this study, 3D structures of quorum-sensing proteins of the Phc pathway (PhcA and PhcR) were computationally modeled, followed by the virtual screening of the natural compounds library against the predicted active site residues of PhcA and PhcR proteins that could be employed in limiting signaling through 3-OH PAME. Two of the best scoring common ligands ZINC000014762512 and ZINC000011865192 for PhcA and PhcR were further analyzed utilizing orbital energies such as HOMO and LUMO, followed by molecular dynamics simulations of the complexes for 100 ns to determine the ligands binding stability. The findings indicate that ZINC000014762512 and ZINC000011865192 may be capable of inhibiting both PhcA and PhcR. We believe that, after further validation, these compounds may have the potential to disrupt bacterial quorum sensing and thus control this devastating phytopathogenic bacterial pathogen.

## 1. Introduction

*Ralstonia solanacearum* (*R. solanacearum*) is known as amongst the deadliest bacterial vascular phytopathogens, with a diverse range of hosts having a wide regional distribution globally, causing substantial yield loss in the tropical, subtropical, and, recently, temperate climate regions too. It is a well-known quarantine pathogen worldwide responsible for enormous agricultural losses [1]. The *R. solanacearum* species complex (RSSC) was grouped mainly into three species, namely *R. solanacearum*, *R. pseudosolanacearum*, and *R. syzygii* subsp. *indonesiensis* [2,3], causing infection to over 400 diverse plant species worldwide and is considered a major challenge to crop production [4,5]. The pathogen spreads through the infested soil and water, enters the host through the plant roots and extensively colonizes the xylem tissues, and produces vascular disorder, causing wilting of the infected Potato, tomatoplants [6,7,8], banana, tobacco, and brinjal are a few of the best examples of crops seriously being affected by vascular bacterial wilt pathogen, *R*. *solanacearum* [9,10]. In south-eastern Louisiana (LA, CA, USA), the yearly yield damage due to bacterial wilt disease in tomatoes ranges between 10 and 50% based on the crop season [11]. Recently, a high disease incidence of 35–40% wilt in tomatoes was reported in Tamilnadu, India [12].

Several plant pathogenic Gram-negative and non-spore-forming bacteria produce small diffusible signaling molecules, for instance, acyl-homoserine lactone (AHL), among their population to communicate with each other, known as quorum sensing (QS) signal molecules [13]. The Phc regulatory system plays a central regulatory and key role that switches to a turn-off mechanism of free-living microorganisms behaviors, which are vital during initial host recognition, development of interaction, biofilm formation, and pathogenesis [14]. RSSC strains utilize the QS system that controls the PhcBSR operon to modulate its virulence to the host plants [15], and to produce and secrete QS signal molecule, methyl 3-hydroxymyristate (3-OH MAME), which accords virulence of the pathogen [16,17]. Similarly, QS also modulates several bacterial functions including gene expression to symbiotic as well as pathogenic relationships with host plants [18]. In addition, *R. solanacearum* QS systems utilize LuxI/LuxR-type regulatory homologs (SolI/SolR) that produce and respond to AHL signal molecules; however, the SolI/SolR system is redundant for virulence [19], whereas anthranilic acid regulates important biological tasks via the production of QS signal molecules, as well as plays dual roles in intra-species signaling and inter-kingdom communication in *R. solanacearum* [20].

The Phc (Phenotype conversion) regulatory mechanism regulates much of the characteristics necessary for infection and virulence in a population density-dependent manner. Exopolysaccharides (EPS) synthesis in *R. solanacearum* is delimited by the PhcA QS system, produced profusely at a higher cell population in culture or during bacterial colonization of host plant xylem tissues [21]. Among the phytopathogenic bacteria, *Ralstonia* has evolved a genus-specific QS system comprising Phc regulatory elements that associate with a distinctive fatty acid derivative signal ‘Phc QS’ [22]. Furthermore, PhcA is also reported to regulate the production of plant cell wall-degrading enzymes, as well as secondary metabolites, namely ralfuranones, which is vital for the virulence of bacteria [17,23]. Likewise, the PhcB and the two-component system genes consisting of PhcS/PhcR constitute an operon in the *R. solanacearum* strain AW1 genome [24]. Moreover, the PhcA QS system actively modulates the expression of ralA and plays a central role that encodes furanone synthase for the production of aryl-furanone secondary metabolites and ralfuranones [16]. The PhcR response regulator in *R*. *solanacearum* post-transcriptionally affects the amount of PhcA, which plays a central role in a multifaceted regulatory pathway, and their activity is regulated by the 3-OH palmitic acid methyl ester (3-OH PAME) QS molecule [25,26]. EPS, pectin methylesterase, and endoglucanase are not produced by PhcA mutants, are hypermotile, and have improved polygalacturonase and siderophore production. Further, transcriptomic analysis of a PhcA mutant grown in tomato xylem vessels showed that PhcA-arbitrated QS may mark the expression of more than 12% of genes in *R. solanacearum*. The production of ralsolamycin molecules possessing an inter-kingdom transmission signal is controlled by the PhcB-dependent QS system in *R. solanacearum* [27]. Similarly, *R. solanacearum* strain OE1-1 produces (R)-methyl 3-hydroxymyristate (3-OH MAME) by way of a QS signal, which is regulated by the PhcB methyltransferase and recognized by the two-component system of PhcS/PhcRQ [28]. By contrast, in RSSC strains, PhcB and PhcS/PhcRQ, concealed by the operon PhcBSRQ, play crucial functions in the Phc QS system [24]. On the other hand, LysR-typ transcriptional regulator (LTTR) PhcA regulates the synthesis and expression of several genes responsible for virulence and other important functions in *R. solanacearum* [15,16]. However, it is reported that Phc QS signal genes that are deficient mutants are known to reduce the RSSC strains’ virulence distinctly in host plants, and the methodology may be useful to inhibit the Phc QS systems [15,17], but the potential to target the Phc QS of RSSC still remains a difficult task [28]. The qualitative detection of proteins by the Immunoblot and Northern test method described that the amount of PhcA was reduced during PhcR post-transcription in *R. solanacearum* via an unspecific mechanism [29].

The molecular structures of the components of the Phc pathway are not known, and therefore the structural implications of 3-OH PAME on QS are also not well explored. In the present study, efforts have been made to generate the 3D structures of Phc pathway quorum-sensing proteins (PhcA and PhcR) and to identify the potential small-molecule inhibitors that could be used by 3-OH PAME to restrain signaling, using state-of-the-art in silico approaches. This study provides an insight into the inhibition or disruption QS mechanism with the identification of potential natural compounds that mimic binding to quorum sensors in *R. solancearum* and, thus, may ultimately help to reduce the vulnerability of the host plant to the devastating phytopathogenic bacterial pathogen, *R. solancearum*.

## 2. Material and Methods

### 2.1. Structure Prediction of PhcA and PhcR

The 3D structures of PhcA and PhcR proteins are still unavailable in the Protein Data Bank (PDB). Therefore, amino acid (aa) sequences of PhcA and PhcR were fetched from the UniProt database. To obtain appropriate templates for homology modeling, the BLASTP [30] search was executed against the PDB database [31] with the consideration of the default parameters. As there was no relevant match in the BLAST search suitable for homology modeling, we proceeded with ab initio protein modeling, fold recognition, and threading approaches using different web servers, viz. QUARK [32], I TASSER [33], and trRosetta [34].

### 2.2. Structure Validation

Using different web servers and tools such as the SAVES server (v6.0, UCLA-DOE LAB, Los Angeles, CA, USA), ProSA [35], and MolProbity (Duke University, Durham, NC, USA), all generated 3D structures of both PhcA and PhcR were further validated. In addition, VADAR (Volume, Area, Dihedral Angle Reporter, University of Alberta, Edmonton, AB, Canada), GeNMR (University of Alberta, Edmonton, AB, Canada), and PROSESS (University of Alberta, Edmonton, AB, Canada) web servers were used to check the Z-score, packaging faults, bump score, gyration radius, and Y angle variance of the models.

### 2.3. Protein Structure Preparation

The validated 3D structures of both PhcA and PhcR were minimized and prepared for further virtual screening and molecular docking studies.

### 2.4. Compound Library Preparation

A library of natural compounds was retrieved in sdf format from the ZINC database [36]. Further, these retrieved natural compounds were imported into ‘Discovery Studio’ (Dassault Systemes BIOVIA, San Diego, CA, USA) and processed using the ‘ligand preparation’ tool.

### 2.5. Virtual Screening

The ligand-binding site in the modeled proteins (PhcA and PhcR) was identified using the 3D Ligand Site program [37]. AutoDock Vina was used for structure-based virtual screening [38]. The molecules were screened using default settings and were scored using Gibbs free energy as implied in Vina. Finally, the top-scored compounds that resulted from the screening were further dealt with using in-depth molecular docking analysis by Glide, Schrödinger [39].

### 2.6. Molecular Docking Study

Glide was used for docking analysis of screened compounds against modeled proteins (PhcA and PhcR) in extra precision (XP) mode [39]. Then, the resulting best-docked protein–ligand complexes were characterized and refined for further analysis based on values of binding energy, intermolecular H–H bonds, and other interactions (hydrophobic and electrostatic). Furthermore, the LigPlot+ and ligand interactions module of Schrödinger were used to show the presence of intermolecular bonds between protein–compound complexes.

### 2.7. Quantum Chemical Calculation

The B3LYP correlation function of density functional theory (DFT) [40] was used to study the reactivity and effectiveness of the screened compounds with antibacterial efficacy against *R. solanacearum* in the form of ‘highest occupied molecular orbital’ (HOMO) and ‘lowest unoccupied molecular orbital’ (LUMO) energies. Whereas ORCA 4.0 [41] was applied to calculate the energy and measurement for the potential drugs, to compute the energy for the potential compounds, the electronic energy, border HOMOs, LUMOs, dipole moment, and gap energy were measured. The following equation was used to calculate the DFT:

$$E=\min_{n}\;\{\int V_{\mathrm{nuclei}}(\vec{r})n(\vec{r})d^{3}\vec{r}+\;F[n(\vec{r})]\}$$

(n ≡ trial density and F ≡ universal functional)

### 2.8. Molecular Dynamics (MD) Simulations

Molecular Dynamics (MD) simulations were conducted for the protein–compound complex to determine the stability and configurational flexibility of all its atoms. We used the Desmond program to perform MD simulations of protein (Apo) as well as protein–compound (Holo) complexes in order to confirm compound binding modalities and present a complete picture of the protein–compound interaction complexes. The top-scoring protein–compound complexes were subjected to a 100-nanosecond (ns) MD simulation. The OPLS4 force field was used to minimize the protein–ligand complexes, and topology and atomic coordinates were obtained automatically. After that, the compound was immersed in an SPC solvent model orthorhombic box (15 × 15 × 10 Å). By adding 0.15 M NaCl, the physiological pH was neutralized. The water box was configured using the Particle Mesh Ewald boundary parameters to warrant that no solute atoms occurred within a 10-angstrom distance of the border. The entire system was simulated at 300 K for 100 ns using the NPT association, and the structural alterations and dynamic characteristics of the proteins were investigated using RMSD and RMSF graphs. The distinction between the foundations of a protein from its primary structural configuration to its final position was measured using RMSD. The RMSF method was employed to find the amenable region of a protein/complex [42]. The interaction diagram of the simulation depicts the most likely compound binding form at the protein’s binding position [43].

## 3. Results and Discussion

### 3.1. Model Generation and Validation

**Figure 1:**
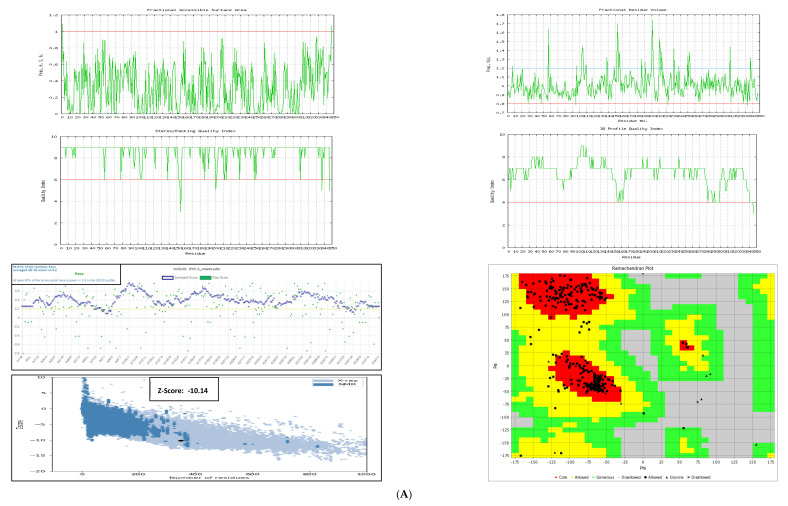

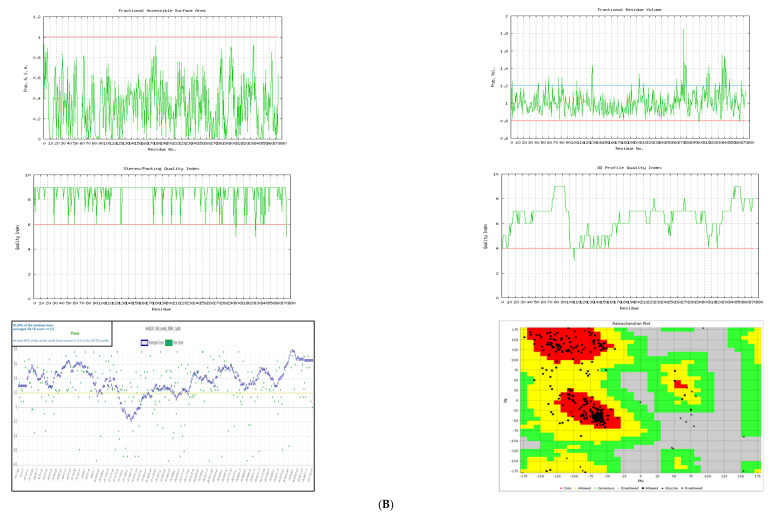
(**A**) Validation of modeled 3D structure of PhcA. (**B**) Validation of modeled 3D structure of PhcR.

In the present investigation, the 3D models of PhcA and PhcR were predicted using state-of-the-art techniques and were validated using different servers and online validation tools (Figure 1A,B). The structure was first evaluated using the Ramachandran plot method and followed by PROCHECK analysis. The models (PhcA and PhcR) were further evaluated with VERIFY 3D with 94.81% PhcA residues and 85.29% PhcR residues exhibited a higher score than 0.2, and which is a very reasonable 3D-1D score for the residues. In addition, the assessment of the functional accessible area, fractional residue volume, stereo/packaging quality, and 3D profile quality index performed by VADAR showed that the residues of both PhcA and PhcR models were within a reasonable range. Based on the above rigorous confirmatory studies, we postulated a putative model for PhcA and PhcR, which was further used for the simulated screening of possible inhibitors PhcPhc. Additionally, we used the CATH database to precisely classify the structures and functions of the PhcA and PhcR binding domains in order to identify other proteins with similarly classified domains and to compare the predicted structure of the PhcA and PhcR binding domains to any known functional analogs. The modeled structures were found to closely match the particular domains of the reference crystal structures (Supplementary Materials, Figures S1 and S2).

### 3.2. Structure-Based Virtual Screening

Auto Dock Vina was employed for the structure-based virtual screening. Active site cavities were assigned as X = −18.400, Y = −36.127, Z = 0.751 for PhcA, and X = −27.186, Y = 23.338, Z = 12.487 for PhcR. The molecules were docked using default settings and were scored using the Gibbs free energy as implied in Vina. The docking scores of the top 10 scoring molecules are given in Table 1 and Table 2 for PhcA and PhcR receptors, respectively. We selected the best common ligand from both (PhcA and PhcR) screening results for more thorough docking interaction analysis, where ZINC000014762512 and ZINC000011865192 for both PhcA and PhcR were found to be the best scoring common ligands/compounds.

### 3.3. Protein–Ligand Interactions of PhcA

**Figure 2:**
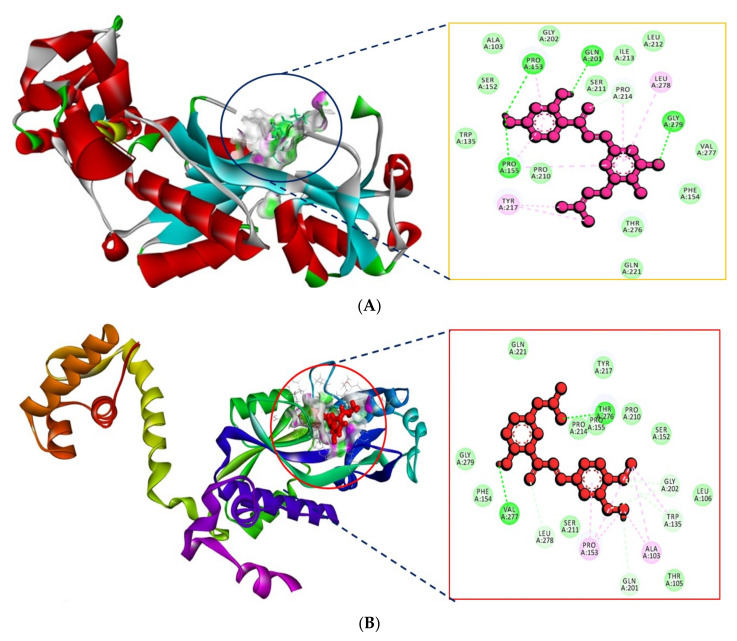
(**A**) Interaction of PhcA with ZINC000014762512. (**B**) Interaction of PhcA with ZINC000011865192.

Table 1 shows the top-scoring 10 molecules against the PhcA receptor. The ligand ZINC000014762512 and ZINC000011865192 were found to have docking scores of −8.7 and −8.3 kcal/mol, respectively, with the active site residues of the PhcA. The hydrophobic part bound in a cavity lined with hydrophobic residues. Specifically, Leu278 and Tyr217 of PhcA were found to be interacting hydrophobically with ZINC000014762512, Pro153, and Ala103 with ZINC000011865192. Hydrogen bond interaction with residues Pro153, Pro155, Gln201, and Gly279 interacted with ZINC000014762512, Val277, and Thr276 with ZINC000011865192. The other docked molecules also showed good binding affinities at PhcA. The interactions of PhcA with ZINC000014762512 and ZINC000011865192 are shown in Figure 2A,B, respectively.

### 3.4. Protein–Ligand Interactions of PhcR Receptor

**Figure 3:**
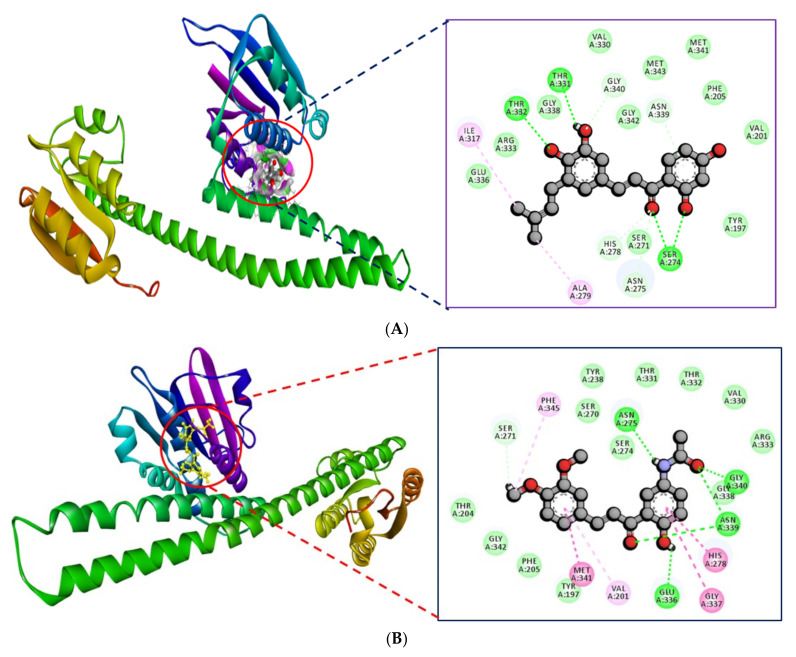
(**A**) Interaction of PhcR with ZINC000014762512. (**B**) Interaction of PhcR with ZINC000011865192.

Table 2 shows the top-scoring 10 molecules against the PhcR receptor. The ligands ZINC000014762512 and ZINC000011865192 were found to have docking scores of −8.5 and −8.6 kcal/mol, respectively, with the active site residues of the PhcR. ZINC000014762512 formed 3 H-bonds with residues Thr332, Thr331, and Ser274, while ZINC000011865192 formed 4 H-bonds with Asn275, Glu336, Asn339, and Gly340 of PhcR. It was observed that ILE317 and ALA279 of PhcR interacted with ZINC000014762512 hydrophobically, while Met341, Val201, Gly337, His278, and Phe345 interacted with ZINC000011865192. The interactions of PhcR with ZINC000014762512 and ZINC000011865192 are shown in Figure 3A,B, respectively.

### 3.5. Molecular Docking by Glide

**Figure 4:**
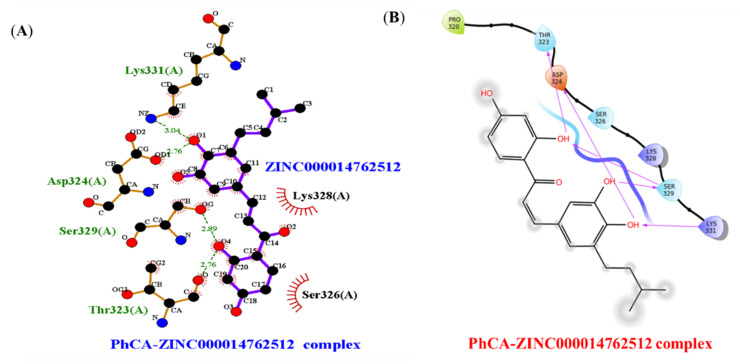
Intermolecular H-bonding, electrostatic, and hydrophobic interactions formed between PhccA–ZINC000014762512 complexes. The image (**A**) is drawn by the LigPlot+ tool and (**B**) ligand interaction module of Schrödinger.

**Figure 5:**
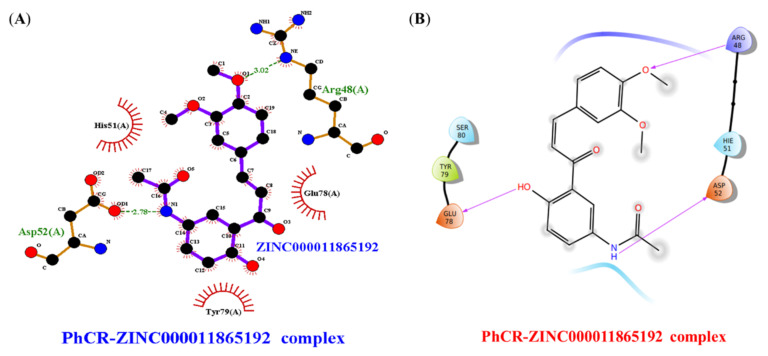
Intermolecular H-bonding, electrostatic, and hydrophobic interactions formed between PhccR–ZINC000011865192 complexes. The image (**A**) is drawn by the LigPlot+ tool and (**B**) ligand interaction module of Schrödinger.

The virtual screening results depicted the compounds/ligands ZINC000014762512 and ZINC000011865192 with better binding affinity against PhcA and PhcR protein targets. Based on the results, the compound ZINC000014762512 was docked against PhcA and ZINC000011865192 with PhcR using Glide. The binding energies of PhcA– ZINC000014762512 and PhcR–ZINC000011865192 interaction complexes are presented in Table 3, Figure 4A,B and Figure 5A,B. The docking data revealed that the binding energy of the ligand–target complexes was varied. Out of the various conformations retained from the docking studies, only the most favorable position with the highest binding energy was carefully chosen for the inter-molecular interaction analysis. The docking analysis reflected the binding energies of −4.120 and −3.312 kcal/mol for PhcA–ZINC000014762512 and PhcR–ZINC000011865192 complexes, respectively.

### 3.6. Quantum Chemical Calculation

**Figure 6:**
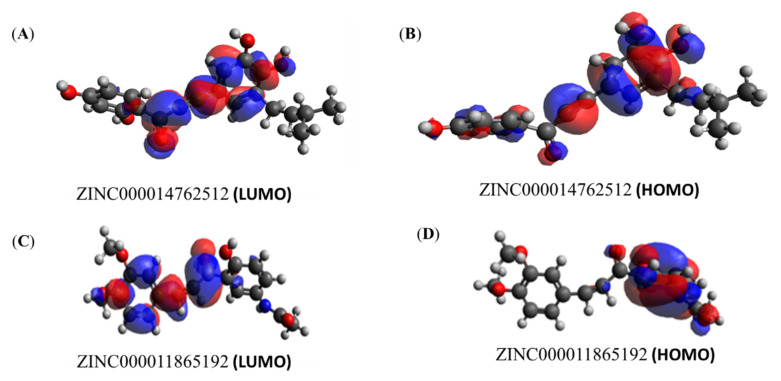
LUMO and HOMO plots of ZINC000014762512 and ZINC000011865192. The color ‘red’ represents positive electron density, while the color ‘blue’ represents negative electron density.

Quantum chemistry was used to investigate the molecular descriptors such as HOMO and LUMO, gap energy, and dipole moment for the ZINC000014762512 and ZINC000011865192 in light of the importance of quantum computation (Table 4). The effective reactivity for ZINC000014762512 and ZINC000011865192 showed the band energy gap (ΔE), i.e., the difference between E_LUMO_ and E_HOMO_, with values 10.048 and 10.199 kcal/mol, respectively. ZINC000014762512 displayed a better reactivity than ZINC000011865192 as it possesses a lower gap energy (Figure 6A–D). Taken together, the compounds ZINC000014762512 and ZINC000011865192 were moved further for MD analysis with PhcA and PhcR proteins.

### 3.7. Analysis of Molecular Dynamics Simulation Trajectory

**Figure 7:**
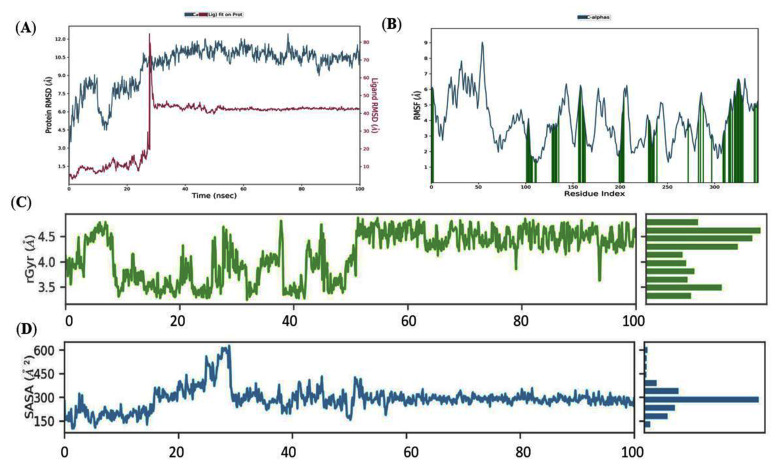
Conformational constancy of ‘Apo’ and ‘Holo’ states of PhcA protein simulation study. (**A**) Backbone-RMSD of PhcA. (**B**) Cα-RMSF profile of PhcA. (**C**) Rg profile of PhcA. (**D**) SASA analysis of Apo and Holo states of PhcA protein throughout the simulations.

**Figure 8:**
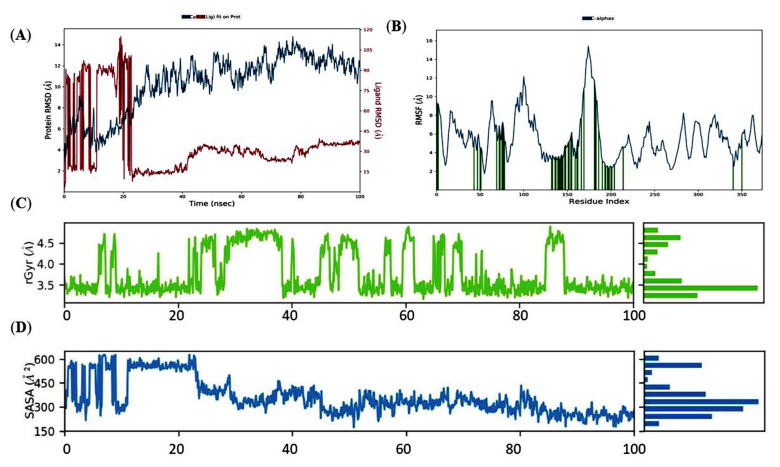
Conformational constancy of ‘Apo’ and ‘Holo’ states of PhcR throughout the simulations. (**A**) Backbone-RMSD of PhcR. (**B**) Cα-RMSF profile. (**C**) Rg profile. (**D**) SASA analysis of Apo and Holo states of PhcR protein throughout the simulations.

Molecular dynamics (MD) is a sophisticated computational tool for predicting and analyzing atoms’ and molecules’ physical movements in the sense of the macromolecular structure-to-function relationship. For a predetermined amount of time, the atoms and molecules were allowed to interact, reflecting the system’s complicated evolution. By contrast, the receptor structural rearrangement and stability of the docked complexes with ZINC000014762512 and ZINC000011865192 were evaluated through a 100 ns MD simulation. The MD simulation of two systems (PhccA and PhccR: Apo states; PhcA-ZINC000014762512 and PhcR-ZINC000011865192 complexes: Holo states) was used to assess the dynamics and stability, RMSD, C-RMSF, Rg, total energy, and SASA using trajectories generated by MD simulations using the Desmond suite of Schrödinger. The dynamic stability of both the complexes PhcA-ZINC000014762512 and PhcR-ZINC000011865192 and its Apo and Holo states were retrieved via RMSD description of the backbone atoms, which were plotted for 100 ns (Figure 7 and Figure 8).

The backbone RMSD profile and graph of the Holo state revealed a stable trajectory after 50 ns of simulation upon comparison to its Apostate. Holo exhibited deviations in the first 50 ns as compared to its Apo state and further reached a stable state. The Apo state represented a noteworthy deviation throughout the MD simulations (1.5–12.1 Å) in comparison to the Holo state with a steady RMSD value between ~6.1 and ~6.2 Å from 50 to 100 ns (Figure 7A). This indicates that ZINC000014762512 can aid in protein stabilization by altering its structure. The RMSD result was then confirmed using RMSF to look at the variation in residues. RMSF plots were used to observe the mobility of distinct residue structures in both phases (Figure 7B).

Overall, the Apo state showed more fluctuations than the Holo state, demonstrating the simulation’s constrained motions. Furthermore, in the Holo state, it was determined that the aa residues between 140 and 150, and 170 and 180 had larger variations in their C atoms than other areas, which might be related to ZINC000014762512’s interaction with the protein. Around 10 terminal residues from both the C- and N-terminal end displayed a greater deviation in all the states that can be ignored. As a result, when ZINC000014762512 and ZINC000011865192 bind, the mobility of residues in the Holo state is reduced compared to the Apo state.

Radius of gyration (rGyr) was used to explain the overall compactness for both the states and stability of ZINC000014762512 in the binding region of the PhcA receptor during the simulation of 100 ns, as shown in Figure 7C. After 50 ns of simulation, rGyr was found to be constant, while the rGyr variation for the ligand ZINC000014762512 in the receptor-binding region of the protein was found to be practically same, ranging from 3.8 to 4.8 and showing stable behavior of the ligand over the 50 ns to 100 ns simulation. This indicates that the Holo state is more compact, indicating that the value of rGyr is inversely proportional to compactness and vice versa. These outcomes are well supported by RMSF analysis.

The hydrophobic interactions reconcile the disclosure of aa to certain solvents. The frequency of these kinds of interactions with the solvent and protein residues is proportional to the surface area under consideration. The outline of SASA (Figure 7D) showed a reduction in the available solvent surface in the Holo state. It is observed that SASA’s detections showed the variation in hydrophilic and hydrophobic interaction regions resulting from the binding of ZINC000014762512, which may potentially change the protein surface orientations by virtue of the aa residue shifting from the accessible area to the hide region. The SASA diagrams of the Holo state described SASA with ~160 to ~300 Å, during the 50 ns to 100 ns MD simulation. This suggests that there might be a change in orientation of the protein surface as a result of the aa residue shift from the attainable area to the enfolded region.

In the case of PhcR-ZINC000011865192, the foundation graph of RMSD of the Holo state released a stable trajectory after 85 ns of simulation upon comparison to its Apo state. In comparison to its Apo state, Holo exhibited aberrations in the first 85 ns before stabilizing. The Apo state depicted a noteworthy deviation throughout the MD simulations (2–14 Å) in comparison to the Holo state, including a stable RMSD value from ~4.1 to ~4.2 Å from 85 to 100 ns (Figure 8A). This depicts that ZINC000011865192 can assist in stabilizing the protein by replacing its conformation. The outcomes of RMSD were later confirmed using RMSF to change the residues. RMSF plots were used to record the mobility of different residues in both phases (Figure 8B). 

It is stated that overall, higher alterations were observed in the Apo than Holo state, which illustrated the restricted movement all around the simulation. By contrast, in the Holo state, it was noticed that the aa residues from 50 to 60, 80 to 110, and 170 to 180 displayed a greater divergence in their Cα atoms compared to other regions, and this could be due to the interaction of ZINC000011865192 with the protein. Around 10 terminal residues from both the C- and N-terminal ends displayed a greater deviation in all the states, which can be ignored. This indicates that the binding of ZINC000011865192 reduces the mobility of residues in the Holo state compared to the Apo state.

The comprehensive compactness to both states and the stability of ZINC000011865192 in the binding area of the PhcR receptor were explained using properties such as rGyr throughout the simulation (Figure 8C). It was obvious from the rGyr variation graphs with respect to the simulation period that rGyr remained steady during the simulation activity after ~85 ns. Similarly, the rGyr distinction for the ligand ZINC000011865192 to the receptor–binding region of the protein was recorded more related as it ranged from 3.2 Å to 3.6 Å and exhibited steady behavior of the ligand during the 80 ns to 100 ns MD simulation. This indicates that the Holo state is more compact, indicating that the value of rGyr is inversely proportional to compactness and vice versa. These outcomes are well supported by RMSF analysis.

The hydrophobic interactions prevent aa from being exposed to certain solvents. The exposed surface area corresponds to the prevalence of these kinds of interactions with the solvent and core protein residues. The diagram of SASA (Figure 8D) revealed a decrease in the ‘accessible solvent surface’ in the Holo state. The binding of ZINC000011865192 impacted the hydrophilic and hydrophobic interaction regions, presumably altering the protein surface orientations due to the aa residue shift from the accessible to the hidden region. The SASA diagram of the Holo state represented SASA with ~160 to ~300 Å, during the 85 ns to 100 ns MD simulation. These findings revealed that there might be changes in orientation on the protein surface because of the aa residue shifts from the accessible area to the hide region.

### 3.8. H-Bond Analysis

**Figure 9:**
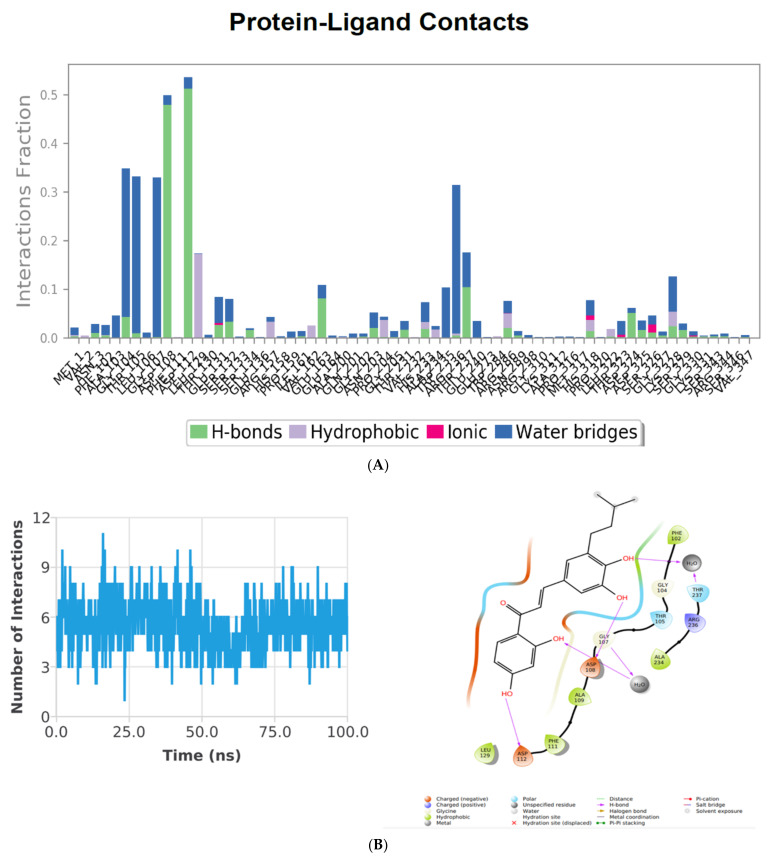
(**A**) Protein ligand contacts plot for heterodimeric PhcA–ZINC000014762512 receptor complex throughout the simulation. (**B**) Blue lines display H-bonds deviation observed in interaction during 100 ns simulation in Holo state. Post-MD simulations of intermolecular H-bonding, electrostatic, and hydrophobic contacts formed between PhcA–ZINC000014762512 complex. The image was drawn by Plot and the ligand interaction module of Schrodinger.

The intermolecular H-bonds of the Holo and Apo were traced during the course of the MD simulations of the PhcA-ZINC000014762512 complex (Figure 9A–C). Throughout the simulation duration, both states (Apo and Holo) reflected a varying number of intermolecular H-bonds.

The stacked bar chart in Figure 9A shows that aa residues such as Ala103, Gly104, Gly107, Asp108, Phe111, Trp235, Arg236, Ala234, and Lys328 play a vital role in the binding as well as regulation of the PhcA protein. Asp108 and Asp112 are the most important aa residues for PhcA protein activity and binding, with the greatest interaction fractions of 0.5 and 0.55, respectively. Because a few protein residues may form several interactions of the similar subtype with the interacting ligand, values above 0.3 are achievable in this histogram. After 100 ns of simulation, the aa residues Asp108 and Asp112 were implicated in creating H-bonds with the ligand. Further, the Holo state simulation represented a diverse number of intermolecular H-bonds up to 85 ns of the simulation (Figure 9B). Two H-bonds (with an average of ~2.051 Å) were represented in the case of the post-MD PhcA–ZINC000014762512 complex (Figure 9C). During simulations, the H-bond-forming residues such as Ser329, Lys331, Asp324, and Thr323 were broken and compensated with novel H-bond (Asp108, and Asp112) residues, van der Waals, and hydrophobic contacts. The findings suggest that the compound, ZINC000014762512, may attain its potentiality against the targeted protein during post-MD simulations.

**Figure 10:**
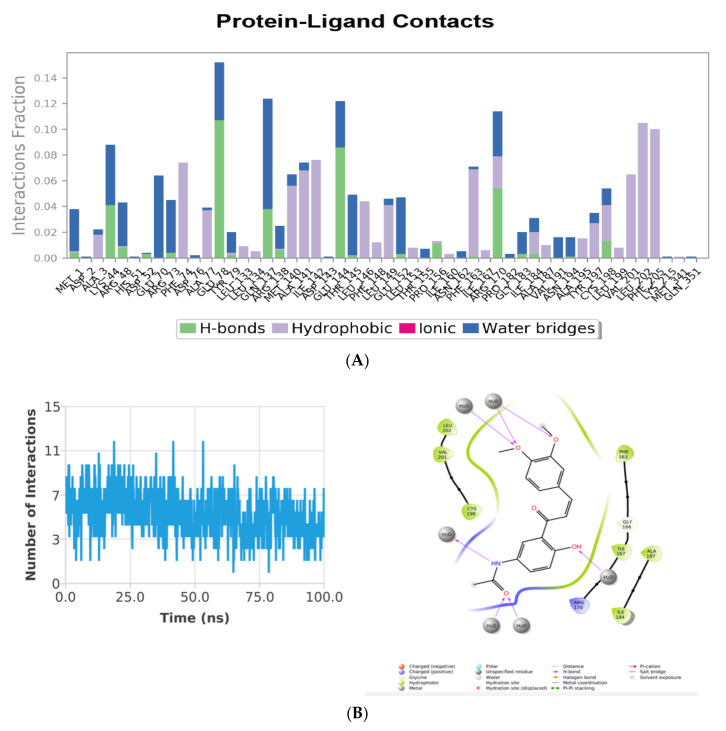
(**A**) Protein ligand contacts plot for heterodimeric PhcR–ZINC000011865192 receptor complex throughout the simulation. (**B**) Blue lines display H-bonds deviation observed in interaction during 100 ns simulation in Holo state. Post-MD simulations intermolecular H-bonding, electrostatic, and hydrophobic contacts made between PhcR–ZINC000011865192 complex. The image was drawn by Plot and the ligand interaction module of Schrodinger.

In case of MD simulation of the PhcR-ZINC000011865192 complex, the aa residues such as Lys44, Glu78, Leu134, Glu137, Glu144, and Arg 170 play a major part in the binding as well as regulation of the PhcR protein, as shown in Figure 10A. On the other hand, Lys44, Glu78, Leu134, Glu144, and Arg170 were the most prominent aa residues for the activity, along with the binding of the PhcR protein as they had the maximum interaction element of 0.09, 0.15, 0.12, 0.12, and 0.11, respectively. By contrast, in the represented histogram, values over 0.08 were possible as certain protein residues could make multiple contacts of the same subtype with the interacting ligand. Glu78 and Glu144 were the aa residues, which were involved in forming H-bonds with the ligand but were broken later during the course of the 100 ns MD simulation. The simulation of the Holo state resulted in a variable number of intermolecular H-bonds up to 85 ns of the simulation (Figure 10B). However, no H-bonds were interpreted in the case of the post-MD PhcA-ZINC000014762512 complex (Figure 10C). During simulations, the H-bond forming residues such as Arg48, Asp52, and Glu78 were broken and compensated with novel van der Waals and hydrophobic contacts. The findings suggest that the compound, ZINC000014762512, may attain its potentiality against the targeted protein during post-MD simulations.

Quorum-sensing (QS) is a communication mechanism and process that allows the bacteria to collectively modify their population behavior with regard to the change in the cell density and species composition, controlling the surrounding microbial community [44]. QS is dependent on the synthesis of low-mass signaling molecules, where the extracellular distribution of these signal molecules is related to the population density of the concerned bacterial species. These signaling molecules may be identified by bacterial cells, enabling the population to take coordinated action after a critical concentration (‘quorum’) has been achieved. Phytopathogenic bacteria are strongly dependent on QS regulation to manage their entry and infection to host plants. The plant pathogenic bacteria employ ‘QS signals’ to modulate various genes, for example, in *R. solanacearum*, for several important functions including epiphytic fitness, motility, EPS production, and exoenzymes production. The plant pathogenic bacteria, *R. solanacearum* species complex (RSSC), causing ’bacterial wilt’ on many crops, uses a QS system for disease development [45], comprising of Phc regulatory functions to mediate its virulence [17]. The vascular wilt pathogen, *R. solanacearum*, controls a virulence-related QS system, the Phc system, that regulates the activity of the LysR-type transcription regulator PhcA [46]. Different master regulators such as PhcA, HrpG, HrpB, and PehR and various two-component regulatory systems such as PhcS/R, PehS/R, VsrA/D, and SolR/I have been well characterized in *R. solanacearum*. *R. solanacaearum* thrives for long periods in the environment and disseminates through surface irrigation and infested soils [47]. The pathogen infection is caused through wounds and natural openings in the host plant root system and becomes systemic after colonization, which develops the characteristic shoot symptoms [48], and the cortex is attacked by the bacteria and pectolytic enzymes disrupt and dissolve the middle lamella of tissues, which enables bacteria to enter through the host tissues, thus releasing nutrients from the host cells along with Hrp effectors. At the end, the plants wilt due to the aggregation and colonization of the bacteria and the EPS in the xylem vessels. Génin et al. 2005 studied the regulatory mechanism of Type III secretion system (Hrp) genes *R. solanacearum* mediated by the global virulence regulator PhcA [22]. Similarly, Delaspre et al. 2007 described that pathogenicity regulator HrpB in *R. solanacearum* induces 3-hydroxy-oxindole synthesis [49]. By contrast, the production of EPS is regulated by PhcA of the QS system, which is a LysR-type transcriptional regulator.

Likewise, the 3-OH PAME signal molecule serves as a divergent two-component regulatory mechanism, which post-transcriptionally modulates PhcA’s activity in *R. solanacearum*. This QS system comprises a membrane-bound sensor-kinase PhcS, which phosphorylates PhcR instead of a DNA-binding domain, which is an abnormal reaction regulator with a C-terminal kinase domain. On the other hand, mutations that directly deactivate the PhcR kinase domain generate a trans dominant allele suppressing the Phc regulon [21]. Therefore, this study indicates that unphosphorylated PhcR is a negative regulator of the Phc phenotype at 3-OH PAME sub-level concentrations. Successive phosphorylation of PhcR in relation to the signal ligand becomes inactivated further [50].

QS is known to regulate the expression of several genes involved in biofilm production, toxin release, exopolysaccharide production, extracellular enzymatic activities, movement, and plasmid replication, among other tasks. In addition to bacterial proliferation, PhcA activity is regulated by the concentrated QS signal molecule, 3-hydroxypalmitic acid methyl ester (3-OH-PAME), or 3-OH-MAME, encoded by PhcB [17]. As a result, QS is essential for the regulatory oversight and production of virulence factors in plant pathogenic bacteria, as well as in subsequent pathogenesis processes [51].

Wet lab experiments have shown that a two-component system (PhcS and PhcR) functions in combination to regulate the expression of PhcA, and modulates PhcA-regulated virulence factors in response to 3-OH PAME [24]. Furthermore, (R)-3-OH MAME) is another newly reported QS-diffusible signal molecule mediating PhcQS in *R. solanacearum* with the involvement of ralfuranones, as its mutant when directly inoculated into tomato xylem vessels was weakly virulent [16]. Recently, the PhcQ signal was reported to play a significant role in the regulation of QS-dependent genes with partial involvement of PhcR in *R. pseudosolanacearum* strain OE1-1 [52]. PhcK, a putative sensor histidine kinase, is now reported to be essentially required for PhcA full expression, leading to the global transcriptional regulator guiding the QS system in *R. solanacearum* strain OE1-1 [53]. Mutant lecM, which otherwise encodes LecM lectin, led to significantly lower ralfuranone, whereas 3-OH MAME is also reported to play a significant role in the QS signaling pathway of *R. solanacearum* strain OE1-1 [54]. Likewise, EPS1 has also been reported to be accompanied with the feedback loop in the QS of *R. solanacearum* strain OE1-1 [54]. Although the adaptation of *R. solanacearum* in resistant tomato cultivar such as Hawaii 7996 takes place by the convergent renovation of the virulence monitoring network, no plant resistance breakdown was reported [55].

Though wet lab research findings are frequently being reported by researchers from across the globe, and few computational investigations too have been initiated (such as virtual screening of quenchers for the signal 3-OH PAME in *R. solanacearum* (AIChE Annual Meeting 2009), no in-depth computational investigation for the identification of potential small molecules mimicking the binding to quorum sensors in *R. solanacearum* has been reported earlier. Therefore, the present investigation was taken up and, for the first time, we reported that common ligands such as ZINC000014762512 and ZINC000011865192 may work as a possible potential inhibitor for both PhcA and PhcR.

## 4. Conclusions

The molecular structures of the components of the Phc pathway are not yet known fully. Therefore, the present effort to model the 3D structure of QS proteins of the Phc pathway and to utilize them for the virtual screening of potential binding partners could be employed in restraining the signaling. PhcA belongs to the LysR family modulating the transcriptional regulator protein that controls the regulation of virulence factors in *R. solanacearum*. Targeting PhcA to reduce or block its expression will be a potential therapeutic approach to combat the pathogenicity of vascular wilt pathogen, *R. solanacearum*. The aim of this present investigation was to gain a better in-depth understanding of the complex physicostructural mechanisms employed by phytopathogenic bacteria, *R. solanacearum*, and to identify the potential novel small molecules that can modulate bacterial growth under plant infection conditions. This study may provide insight to disrupt bacterial QS by utilizing natural compounds ZINC000014762512 and ZINC000011865192 capable of inhibiting both PhcA and PhcR and ultimately reducing the vulnerability of this devastating plant pathogenic bacterium.

## Figures and Tables

**Table 1:** Top 10 scoring molecules against PhcA receptor.

Ligand	Affinity (kcal/mol)
**ZINC000014762512**	**−8.7**
ZINC000014612777	−8.5
ZINC000005175329	−8.4
ZINC000095919156	−8.4
**ZINC000011865192**	**−8.3**
ZINC000005158606	−8.3
ZINC000012447533	−8.3
ZINC000014762500	−8.3
ZINC000085510993	−8.3
ZINC000004252711	−8.2

In bold are the common ligands for both PhcA and PhcR.

**Table 2:** Top 10 scoring molecules against PhcR receptor.

Ligand	Affinity (kcal/mol)
ZINC000012296302	−8.9
ZINC000095485992	−8.8
**ZINC000011865192**	**−8.6**
ZINC000095919156	−8.6
ZINC000095919158	−8.6
**ZINC000014762512**	**−8.5**
ZINC000031167012	−8.5
ZINC000004695648	−8.4
ZINC000014780728	−8.4
ZINC000014762500	−8.3

In bold are the common ligands for both PhcA and PhcR.

**Table 3:** Molecular docking of ZINC000014762512 and ZINC000011865192 with PhcA and PhcR using Glide.

Sl. No.	Target	ZINC ID	Binding Energy (kcal/mol)	No. of H-Bonds	H-Bond Forming Residues	Average Distance Of H-Bonds (Å)
1.	PhccA	ZINC000014762512	−4.120	6	SER329, LYS331, ASP324, THR323	~2.097
2.	PhcR	ZINC000011865192	−3.312	3	ARG48, GLU78,ASP52	~1.972

**Table 4:** Electronic energy, energy in HOMO, LUMO, gap energy, and dipole moment of ZINC000014762512 and ZINC000011865192.

Compound ID	Electronic Energy (eV)	E_LUMO_ (kcal/mol)	E_HOMO_ (kcal/mol)	GAP Energy (∆E) (kcal/mol)	Dipole Moment (Debye)
ZINC000014762512	−31079.373	2.208	−7.840	10.048	5.98349
ZINC000011865192	−31517.114	1.958	−8.241	10.199	1.80864

## Data Availability

The data presented in this study are available in this article and the accompanying Supplementary Materials.

## Supplementary Materials

The following supporting information can be downloaded at: https://www.mdpi.com/article/10.3390/molecules27093034/s1, Figure S1: Phc A (Blue color) superimposed with matched reference structure 1(UTH) (cyan color); Figure S2: Phc R (Blue color) superimposed with matched reference structure 5idm (cyan color).
