# Supplementary material for: Biocomputational Assessment of Natural Compounds as a Potent Inhibitor to Quorum Sensors in Ralstonia solanacearum
Source: Molecules. 2022 May 9;27(9):3034. doi: 10.3390/molecules27093034 (PMC9102662; doi:10.3390/molecules27093034)
Supplement: Supplementary file 1 [file molecules-27-03034-s001.zip › molecules-1688692-supplementary.pdf]

Supplementary Materials

Biocomputational Assessment of Natural Compounds as a Potent Inhibitor to Quorum Sensors in *Ralstonia solanacearum*

Sunil Kumar [1,2,*], Khurshid Ahmad [1], Santosh Kumar Behera [3], Dipak T. Nagrale [4], Anurag Chaurasia [5], Manoj Kumar Yadav [6], Sneha Murmu [2], Yachana Jha [7], Mahendra Vikram Singh Rajawat [1], Deepti Malviya [1], Udai B. Singh [1], Raja Shankar [8], Minaketan Tripathy [9] and Harsh Vardhan Singh [1,*]

[1] ICAR-National Bureau of Agriculturally Important Microorganisms, Mau 275103, India; ahmadk@ynu.ac.kr (K.A.); rajawat.mvs@gmail.com (M.V.S.R.); deeptimalviya77@gmail.com (D.M.); nbaimudai@gmail.com (U.B.S.)
[2] ICAR-Indian Agricultural Statistics Research Institute, New Delhi 110012, India; murmu.sneha22@gmail.com
[3] National Institute of Pharmaceutical Education and Research, Ahmedabad 382355, India; bioinfo.santosh@gmail.com
[4] ICAR-Central Institute for Cotton Research, Nagpur 440010, India; dip29unique@gmail.com
[5] ICAR- Indian Institute of Vegetable Research, Varanasi 221305, India; govtofindia.icar@gmail.com
[6] Department of Bioinformatics, SRM University, Sonepat 131029, India; manojiids@gmail.com
[7] N. V. Patel College of Pure and Applied Sciences, S.P. University, Anand 388315, India; yachanajha@ymail.com
[8] ICAR-IIHR, Hessaraghatta Lake Post, Bengaluru 560089, India; rajascientists@gmail.com
[9] Department of Pharmacy, Sitaram Kashyap College of Pharmacy, Rahod, Chhattisgarh 495556, India; minaketantripathy@gmail.com
* Correspondence: skybiotech@gmail.com (S.K.); drharsh2006@rediffmail.com (H.V.S.)

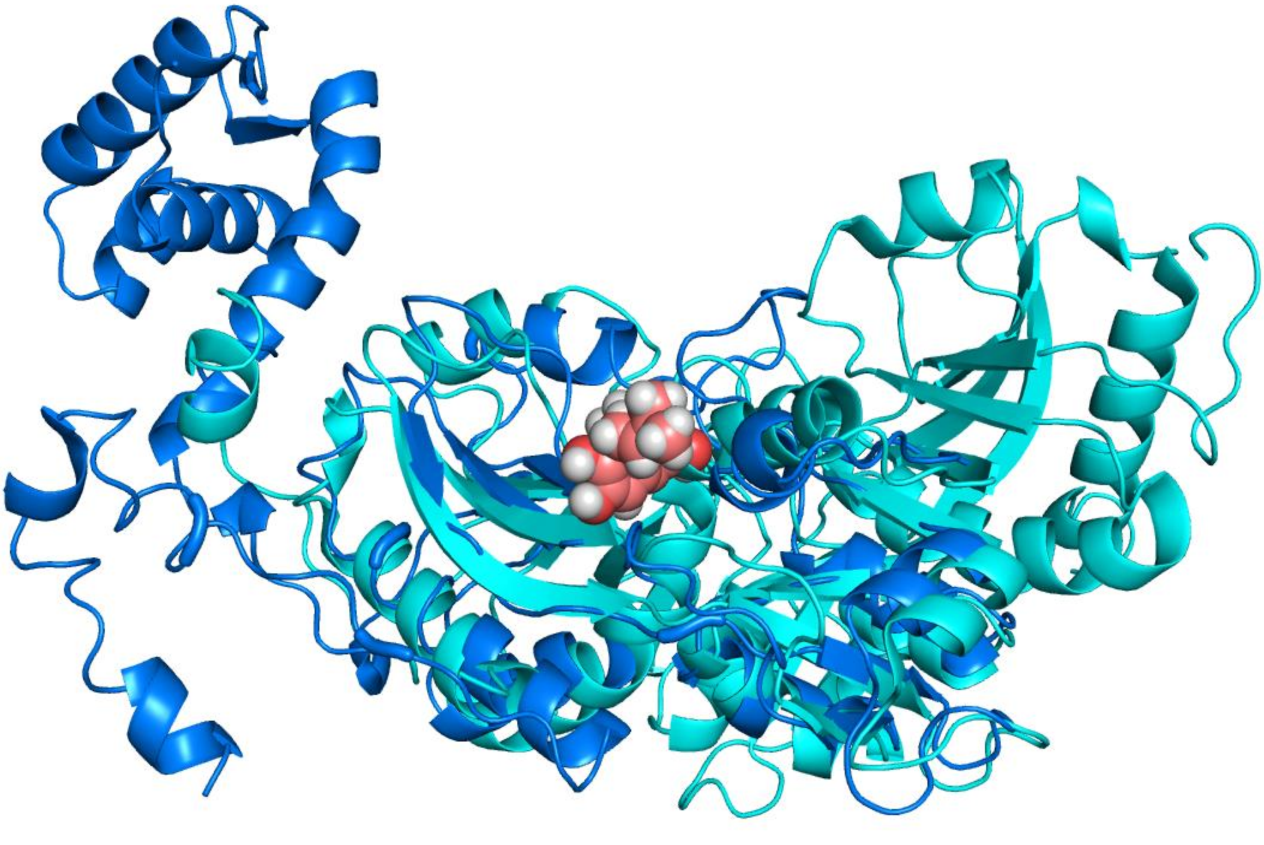

Figure S1. Phc A (Blue color) superimposed with matched reference structure 1(UTH) (cyan color).

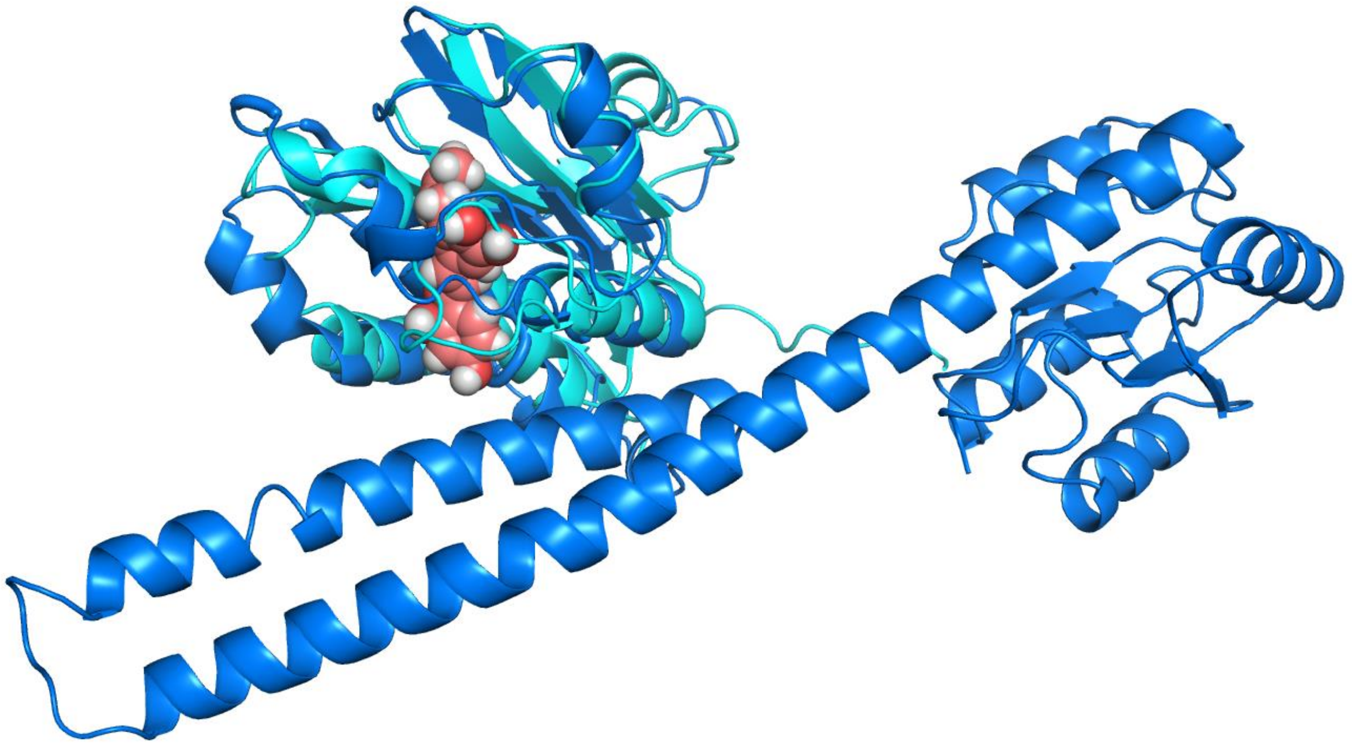

Figure S2. Phc R (Blue color) superimposed with matched reference structure 5idm (cyan color).
